# A universal approach for drainage basins

**DOI:** 10.1038/s41598-019-46165-0

**Published:** 2019-07-08

**Authors:** Erneson A. Oliveira, Rilder S. Pires, Rubens S. Oliveira, Vasco Furtado, Hans J. Herrmann, José S. Andrade

**Affiliations:** 1 Programa de Pós Graduacção em Informática Aplicada, Universidade de Fortaleza, 60811-905 Fortaleza, Ceará Brazil; 2 Mestrado Profissional em Ciências da Cidade, Universidade de Fortaleza, 60811-905 Fortaleza, Ceará Brazil; 3 Departamento de Física, Universidade Federal do Ceará, Campus do Pici, 60451-970 Fortaleza, Ceará Brazil; 4 PMMH, ESPCI, 7 quai St Bernard, 75005 Paris, France; 5 ETH Zürich, Computational Physics for Engineering Materials, Institute for Building Materials, Wolfgang-Pauli-Strasse 27, Hit CH-8093 Zürich, Switzerland

**Keywords:** Statistical physics, thermodynamics and nonlinear dynamics, Hydrology

## Abstract

Drainage basins are essential to Geohydrology and Biodiversity. Defining those regions in a simple, robust and efficient way is a constant challenge in Earth Science. Here, we introduce a model to delineate multiple drainage basins through an extension of the Invasion Percolation-Based Algorithm (IPBA). In order to prove the potential of our approach, we apply it to real and artificial datasets. We observe that the perimeter and area distributions of basins and anti-basins display long tails extending over several orders of magnitude and following approximately power-law behaviors. Moreover, the exponents of these power laws depend on spatial correlations and are invariant under the landscape orientation, not only for terrestrial, but lunar and martian landscapes. The terrestrial and martian results are statistically identical, which suggests that a hypothetical martian river would present similarity to the terrestrial rivers. Finally, we propose a theoretical value for the Hack’s exponent based on the fractal dimension of watersheds, γ = D/2. We measure γ = 0.54 ± 0.01 for Earth, which is close to our estimation of γ ≈ 0.55. Our study suggests that Hack’s law can have its origin purely in the maximum and minimum lines of the landscapes.

## Introduction

Drainage basins play a fundamental role in the hydrologic cycle, which make them essential to diversity and maintenance of Life on Earth^1^. The longstanding problem of characterising drainage basins has drawn much attention due to its importance in a variety of environmental issues, such as water management^2–4^, landslide and flood prevention^5–9^, and aquatic dead zones^10–12^. In this context, *drainage basins*, or simply *basins*, are all land areas sloping toward a single outlet, *e.g*. a river mouth or points of higher infiltration or evaporation rates. They are outlined by abstract boundary lines, called *topographic divides* or *watersheds*. The concept of watersheds appears in many other seemingly unrelated areas like percolation theory^13,14^, image segmentation and medicine^15–18^, and even international borders^19,20^.

Watersheds are recognized as fractals in several cases^21–24^, exhibiting self-similarity and a well defined *fractal dimension*. There are several objects that share the same fractal dimension of watersheds on uncorrelated random substrates (*viz. D* ≈ 1.22), such as optimal paths under strong disorder^25–28^, minimum spanning trees on random networks^29^, backbones of the optimal path crack^30–32^, and bridge bonds on ranked surfaces^33^. All these loopless paths belong to the same universality class as watersheds. Furthermore, it is also known that watersheds are Schramm-Loewner evolution curves^34^ and that it is possible to define hydrological watersheds^35^, where the infiltration process in the soil is taken into account.

The availability of digital elevation models (DEMs) allowed the development of modern techniques to automatically delineate watersheds. Nowadays, these methods are used in the standard Geographic Information System (GIS) software and are well established as a fundamental tool in Geoscience^36^. The basic idea behind these methods is the calculation of the flow directions, which can be defined in several ways depending on the assumptions on the flow and number of neighbouring cells in the grid^37–44^. Simultaneously to the development of these methods, the concept of watersheds were also introduced in the context of contour delineation^45^ and became a widely used tool for image processing. The key difference among these methods is that those adopted in image processing do not use channels to define watershed lines. Instead, they simply search the landscape for the highest lines that divide it. In this context, even the most modern computational models are not able to get all watersheds of the globe at once, they are usually limited to specific regions. As compared to all these methods, methods based on invasion percolation as ours do not need to explore the entire space but only a fractal subset of it. Therefore, our method becomes more and more efficient the larger the landscape.

Here, we propose a simple model to fully delineate multiple drainage basins for any given landscape based on the traditional Invasion Percolation (IP) model^46^, which is known to be a Self-Organized Criticality (SOC) model^47^, *i.e*. the IP model does not need a tuning parameter to evolve and identify the watershed. Our method allows us to study the morphological segmentation of global maxima and minima in image processing, even if the landscape stands for the gray scale of a brain Magnetic Resonance Imaging (MRI) or the heights of a celestial body with or without river channels. The novelty of our approach is to characterize all basins from a single height dataset through the definition of a reference (sea) level, *i.e*. our approach is free of parameter tuning.

## The Model

In 2009, Fehr *et al*.^22–24^ introduced a model, called Invasion Percolation-Based Algorithm (IPBA), in order to extract watersheds from landscapes. The IPBA was proposed for a regular square lattice of size *L* with fixed boundary conditions in the vertical direction and periodic boundary conditions in the horizontal direction, where the height of each site *i* was represented by *h*_*i*_. It was also defined that the upper and lower lines of the lattice represent the sinks of two basins, *e.g*. one at the North (N) and other at the South (S), respectively. In this context, the following rule for the identification of the basins was proposed: For each site *i*, one applies the IP model, defining that the basin (N or S) to which the site *i* belongs is the one that the IP invaded cluster reaches first. Thus, all sites of the lattice belong to one of the two basins and the interface line between them defines the watershed. To improve the computational performance of the task of finding the interface line, an efficient sweeping strategy was also introduced: (*i*) Initially, the sites are chosen along a straight line that connects the sinks. Therefore, when the IP processes from two neighbouring sites evolve to different sinks, a segment of the watershed lies between them. (*ii*) From then on, the sites are chosen only in the neighbourhood of the already known watershed segments in order to reveal more segments of the watershed, resulting at the end in the complete watershed. Fehr *et al*.^22^ also showed that the IPBA follows the same dynamics as the Vincent-Soille algorithm^15^. The Vincent-Soille algorithm has a direct interpretation, which is basically a flooding process, although it is computationally inefficient. The main advantage of the IPBA is its computational performance, the IPBA presents a sublinear complexity time since it only explores a fractal subset of space of fractal dimension 91/48^22^ and this characteristic allows us to perform a global analysis to the drainage basins.

**Figure 1:**
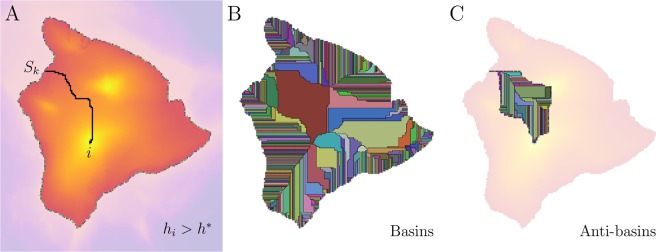
Extension of the Invasion Percolation-Based Algorithm (IPBA). We use a Digital Elevation Model (DEM) from the Island of Hawaii’s region to show the three steps of our approach. (**A**) Single height cluster composing by all sites with *h*_*i*_ > h^*^, where h^*^ = 0 (sea level) throughout the study. The landscape colour is arranged from dark purple (small heights) to light yellow (large heights) in linear scale. We represent in different colours all sinks, namely, the sites that are in the height cluster border, in this case, the sea shore. Finally, the Invasion Percolation (IP) cluster of site *i* is shown in black. The IP process starts at the site *i* and finishes at the sink *S*_*k*_. (**B**) All drainage basins identified with our algorithm. The basins have the colours of their sinks and the black lines stand for the watershed network. (**C**) All drainage anti-basins from the largest drainage basin. The anti-basins are also represented by several colours and the black lines, in this case, stand for the anti-watershed network.

Our aim is to define a robust mathematical model for the delineation of multiple drainage basins through an extension of the IPBA. Suppose a regular rectangular lattice *L*_*x*_ × *L*_*y*_, where the height of each site *i* is *h*_*i*_, analogous to the original model. We introduce a height threshold h^*^ such that, if *h*_*i*_ > h^*^, then the *i* th site belongs to a cluster, which we call *height cluster*, composed by all connected sites with height above that threshold. Otherwise, the *i* th site does not belong to any cluster. As explained in the following section, we adopted h^*^ = 0 throughout this study, which for Earth corresponds to sea level. For this particular choice, the height clusters define continents and islands on Earth, as shown in Fig. 1A. Here, the sinks *S*_*k*_ (*k* = 1, 2, …, *N*_*b*_) are all the *N*_*b*_ border sites of the height clusters, *i.e*. the sea shore on Earth. Consequently, we know *a priori* that they define *N*_*b*_ drainage basins separated by several interface lines, but their specific sizes and shapes need to be determined. Similarly to the ideas proposed by Fehr *et al*.^22^, we define the following rule to identify basins present in the height clusters: The IP model is applied for each site *i* defining that the basin (*S*_*k*_) at which the site *i* belongs is the one that the IP invaded cluster reaches first (see Fig. 1A). As depicted in Fig. 1B, the set of interface lines forms the *watershed network* that separates all basins in the height clusters. We also use a strategy analogous to the original IPBA to improve the performance of finding the watershed network. Here, the sweeping occurs in each basin *S*_*k*_ as follows: (*i*) The sink *S*_*k*_ defines the ends (initial and final segments) of its yet not identified watershed. (*ii*) For each basin, the sweeping occurs only at sites neighboring the already known watershed segments in order to reveal the missing ones. In other words, we scanned the sites along the watershed inner perimeter neighbourhood of each basin. This strategy drastically reduces the number of times that we need to apply the IP algorithm for the identification of the watershed network. (*iii*) Optionally, a simple burning algorithm can be applied to each basin in order to evaluate its area^47^.

Actually, we consider two versions of our algorithm along the study: A version with traditional periodic boundary conditions in horizontal direction and unconventional periodic boundary conditions in vertical direction for real landscapes, and another version with fixed boundary conditions on both directions for artificial landscapes. The unconventional periodic boundary conditions, adopted for real landscapes, are defined by imposing that each site in the top (bottom) row be neighbour of every other site in the top (bottom) row. These boundary conditions represent a mapping of a sphere into a lattice. They are needed because we performed all measures on the entire globe for real landscapes.

A natural extension of the watershed concept is the definition of its reciprocal line, called anti-watershed. The *anti-watersheds* are composed by lines of minimal heights, in contrast to the watersheds, defined in terms of lines of maximal heights. Therefore, if the basins can (grossly) be understood as “cavities” in a surface, then the anti-basins can also be understood as “humps” on that same (inverted) surface. We emphasize that anti-watersheds do not necessarily represent river channels, since a river channel (or a channel) is a “clearly defined watercourse (“natural or man-made channel through or along which water may flow”) which periodically or continuously contains moving water”, or is a “watercourse forming a connecting link between two water bodies”, or even is the “deepest portion of a watercourse, in which the main stream flows”^48^, while a anti-watershed line may never contain any flowing water. Given an upside-down landscape, the same approach for watersheds can be considered to define the anti-watersheds. In this case, the watershed network represents the *anti-watershed network* in the original landscape. Furthermore, we can also define an anti-watershed network within a drainage basin (see Fig. 1C). Here, the lines of minimal heights represent the most deeper rivers and their tributaries in a lot of situations.

We highlight that our model just takes into account the drainage basins that flow out to the oceans, *i.e*. we are ignoring the *endorheic drainage basins* (or simply *endorheic basins*), which are those basins that flow out to any place other than the oceans, *e.g*. lakes or swamps, their amounts of water being balanced by infiltration or evaporation^49^. Nonetheless, we could also include such basins in our model introducing additional sinks ${S^{\prime} }_{k}$ (where *k* = 1, 2, …, *N*_*e*_ and *N*_*e*_ is the number of endorheic basins), located at the points of higher evaporation rates, for example. In this context, the total number of drainage basins (*N*_*b*_ + *N*_*e*_) would depend on the spatial resolution of the data and the information available about the endorheic basins. Our code is available at https://github.com/erneson.

## Results

**Figure 2:**
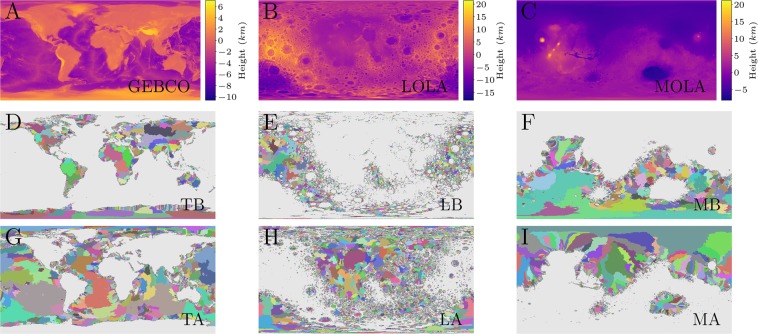
Landscape, basins and anti-basins for Earth, Moon, and Mars. (**A**) General Bathymetric Chart of the Oceans (GEBCO)^50^. (**B**) Lunar Orbiter Laser Altimeter (LOLA)^51^. (**C**) Mars Orbiter Laser Altimeter (MOLA)^52^. In all three cases, the heights are in units of kilometre (*km*) and represented in linear scale. (**D**–**F**) Drainage basins for heights above h^*^ of the GEBCO, LOLA, and MOLA landscapes. (**G**–**I**) Drainage anti-basins for heights below h^*^ of the GEBCO, LOLA, and MOLA landscapes. Note that h^*^ = 0 (sea level for Earth and hypothetical sea level for Moon and Mars) throughout the study. Here, we use the following abbreviations: Terrestrial Basins (TB), Terrestrial Anti-basins (TA), Lunar Basins (LB), Lunar Anti-basins (LA), Martian Basins (MB), and Martian Anti-basins (MA). Sites below the height threshold h^*^ are shown in grey.

**Figure 3:**
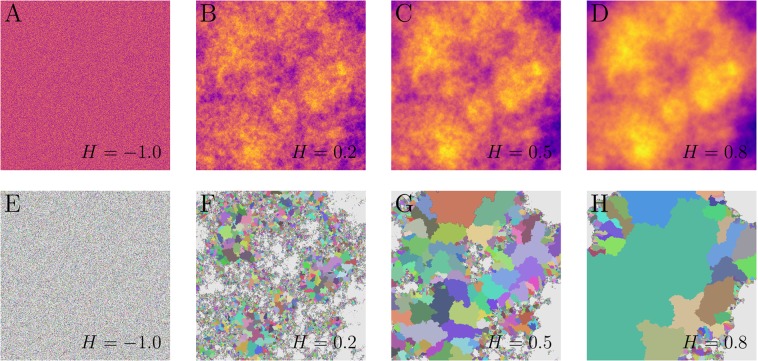
Artificial landscapes and their respective basins. Fractional Brownian motion (fBm) landscapes generated by the Fourier filtering method (Ffm) for four regimes. A typical uncorrelated landscape (*H* = −1) is shown in (**A**), a landscape generated with negative correlations (*H* = 0.2) in (**B**), a Brownian motion landscape (*H* = 0.5) in (**C**), and a landscape with positive correlations (*H* = 0.8) in (**D**). All fBm landscapes shown share the same size *L* = 1024 and the same random seed. The heights are represented in linear scale, where lighter (darker) colours stand for higher (lower) heights. The basins defined by our approach corresponding to the fBm landscapes shown in (**A**–**D**) are shown in (**E**–**H**), respectively. Sites below the height threshold h^*^ are show in grey.

We applied the IPBA model to real and artificial landscapes in order to study the statistical properties of the drainage basins on Earth, Moon, and Mars. For real landscapes, we use three different DEMs throughout this study: the General Bathymetric Chart of the Oceans (GEBCO)^50^, the Lunar Orbiter Laser Altimeter (LOLA)^51^, and the Mars Orbiter Laser Altimeter (MOLA)^52^. Such datasets consist of the map of heights for the Earth, Moon and Mars, respectively. Moreover, we obtained the artificial landscapes through the *fractional Brownian motion* (fBm)^53^. We chose this method because we are interested in generating landscapes with tunable spatial long-range correlations since it is known that such correlations change the statistical properties of watersheds^24^. We show all landscapes in Figs 2A–C and 3A–D. We considered two scenarios for GEBCO, LOLA, and MOLA datasets: the original and the upside-down landscape orientations. We emphasize that we chose the GEBCO dataset to represent the terrestrial landscapes because it includes altimetric and bathymetric heights, which makes it more similar to LOLA and MOLA datasets. In other words, we used the GEBCO dataset in order to make a general and uniform comparison between Earth and two celestial bodies that do not have oceans (Moon and Mars). To perform a global analysis, the resolutions of the real datasets were decreased by a factor of *r* = 8, where *r* is the resolution factor. Thus, each tile of 8 × 8 sites was replaced by a single site with a value given by the mean of all 64 original sites. The sizes of the used lattices were 5400 × 2700 for GEBCO (original 43200 × 21600), 5760 × 2880 for LOLA (original 46080 × 23040), and 5760 × 2880 for MOLA (original 46080 × 23040). For the fBm landscapes, we considered only the original orientation due to the natural symmetry of the Gaussian distribution used in the Ffm. In this case, we averaged our simulations over 10 samples of lattices of 4096 × 4096 for the traditional range of the Hurst exponent *H* (0 ≤ *H* ≤ 1). The following subsections show the corresponding results.

### Real landscapes

**Figure 4:**
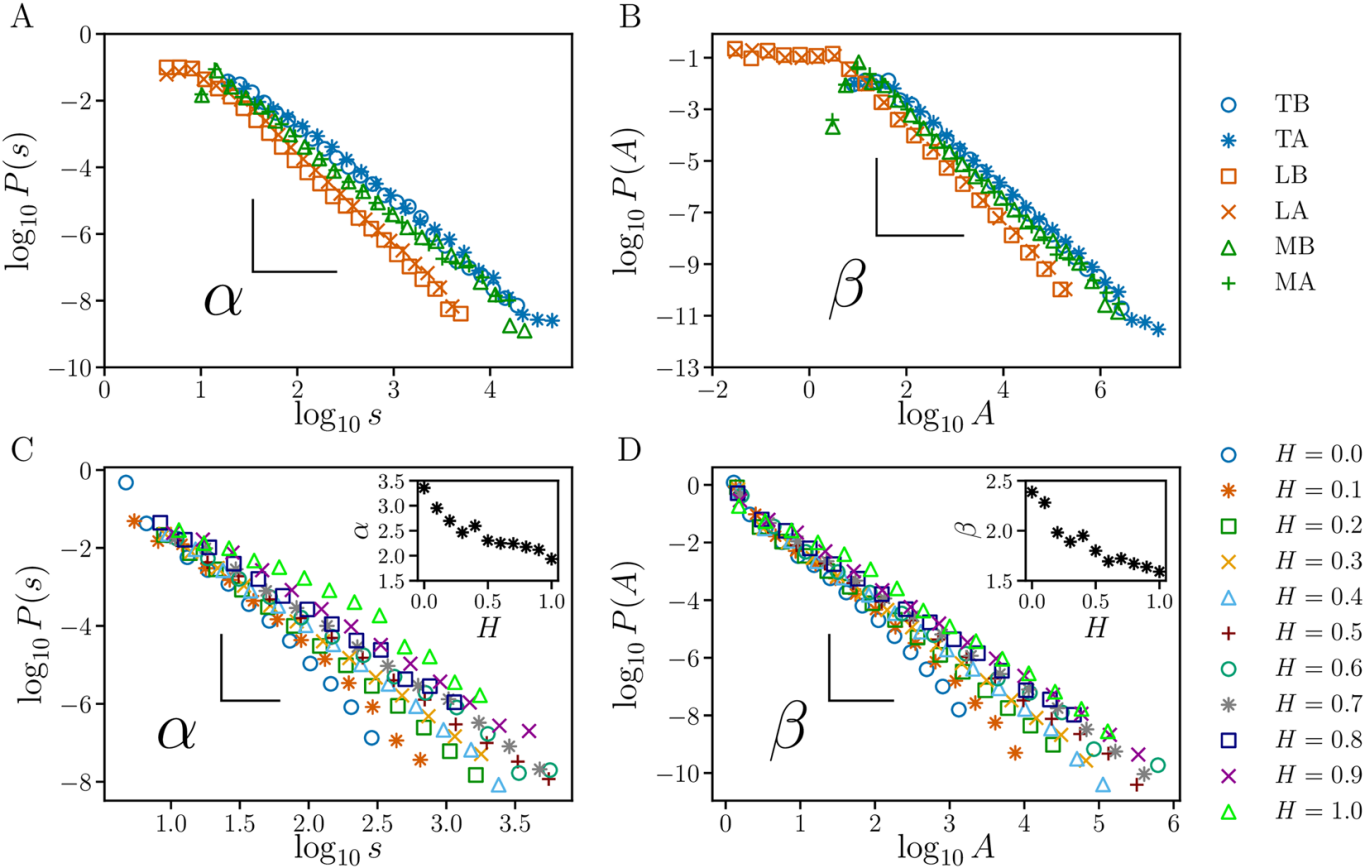
Log-log plots of the perimeter and area distributions for basins and anti-basins on real and artificial landscapes. (**A**) The perimeter distributions for basins and anti-basins on Earth, Moon and Mars. (**B**) The surface area distributions for basins and anti-basins on Earth, Moon and Mars. In both, we use the following abbreviations: Terrestrial Basins (TB), Terrestrial Anti-basins (TA), Lunar Basins (LB), Lunar Anti-basins (LA), Martian Basins (MB), and Martian Anti-basins (MA). We show all exponents for real landscapes in Table 1. (**C**) The perimeter distributions for several values of the Hurst exponent *H*. (**D**) The surface area distributions for several values of *H*. The insets show the behaviour of the exponents *α* and *β* in relation to *H*. All exponents are calculated through the Ordinary Least Square (OLS) fits^54^. We also obtain similar exponents using a Maximum Likelihood Estimator (MLE)^55^ (See SI).

Here, we show the main results of our approach applied to real landscapes. In Fig. 2D,G, we show the results of our model applied to the original and upside-down landscapes of the Earth. In Fig. 2E,H,F and I, we use the height threshold h^*^ = 0 (hypothetical sea level) and perform the same analysis used for Earth’s landscape to obtain the basins and anti-basins for the Moon and Mars. We note that the basins and anti-basins look similar in the terrestrial and martian landscapes, while in the lunar landscape, they are affected by the *impact basins*, *i.e*. craters originated from the impact of asteroids. This similarity is quantified here through the statistical distributions of perimeters and areas of the basins and anti-basins for Earth, Moon and Mars. We included the lunar analysis as a counterpoint to show that the observed similarity between the terrestrial and Martian results is indeed genuine. The log-log plots of all these distributions shown in Fig. 4A–B clearly indicate the presence of long tails extending over several orders of magnitude. Moreover, these tails approximately follow power-law behaviors, *P*(*s*) ∝ *s*^*α*^ and *P*(*A*) ∝ *A*^*β*^, for the perimeters and areas, respectively. By performing Ordinary Least Square (OLS) fits^54^ to the corresponding distributions, we obtained estimates for the power-law exponents *α* and *β* that are summarized in Table 1. Similar results where found using a Maximum Likelihood Estimator (MLE)^55^, as shown in the Supplementary Information (SI).

**Table 1:** Exponents α and β for real landscapes.

Cases	Abbreviation	*α*	*β*
Terrestrial Basins	TB	2.30 ± 0.03	1.74 ± 0.02
Terrestrial Anti-basins	TA	2.26 ± 0.03	1.72 ± 0.02
Lunar Basins	LB	2.70 ± 0.02	1.93 ± 0.01
Lunar Anti-basins	LA	2.64 ± 0.04	1.89 ± 0.02
Martian Basins	MB	2.33 ± 0.04	1.75 ± 0.02
Martian Anti-basins	MA	2.26 ± 0.02	1.73 ± 0.02

These exponents were determined through the Ordinary Least Square (OLS) fits^54^ to the corresponding distributions. Similar results were found using a Maximum Likelihood Estimator (MLE)^55^ (See SI).

We found that the terrestrial and martian results are statistically identical, which suggests the surfaces of both planets underwent a similar formation history and that a hypothetical martian river would present some level of similarity to the terrestrial rivers, since both landscapes share the same statistics for watershed (maxima) and anti-watershed (minima) lines. There is already evidence that the Martian channels were formed by a series of fluvial and non-fluvial processes^56^. Furthermore, we found evidence that the obtained perimeter and area exponents are independent of the resolution factor *r* (See SI). It is known that the effects of finite size are attenuated as the system size increases. We also obtained similar results for other values of h^*^ on Earth, Moon and Mars.

**Figure 5:**
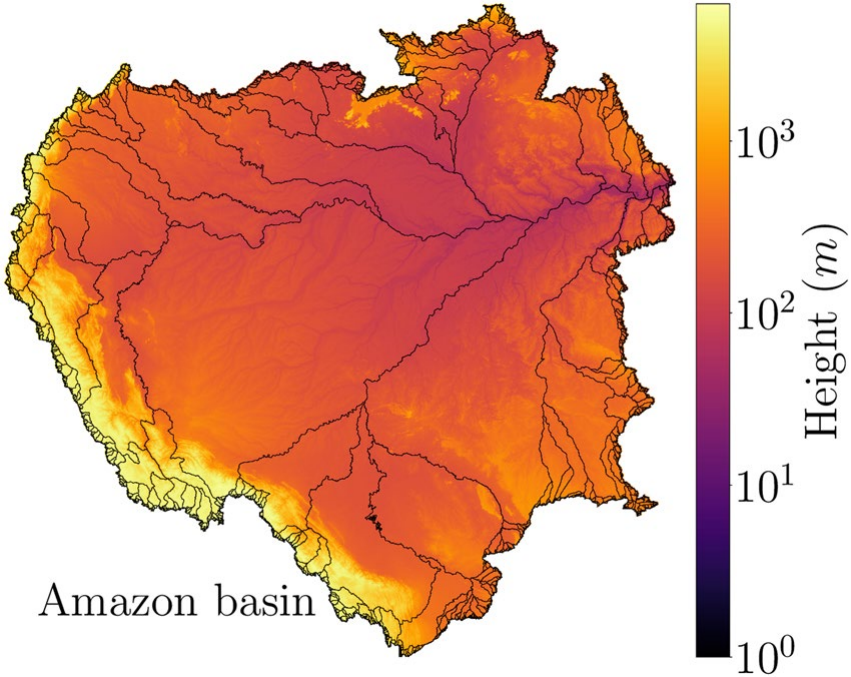
The Amazon basin. The drainage basin of the Amazon region obtained by our algorithm. The heights are represented in metres (*m*) and in logarithmic scale in order to show the details of the Andes mountain range and the Amazon river. The black lines stand for the anti-watershed network, which shows an impressive similarity with the Amazon river network.

In Fig. 5, we show the Amazon basin and its associated anti-basins defined by our algorithm on GEBCO dataset at the original resolution. In this special case, we are removing all internal sinks from the South American continent, *i.e*. we are allowing the existence of sites with negative heights within South America. We emphasise that several rivers (the deeper ones) follow the anti-watershed lines. The black lines in Fig. 5 stand for the anti-watershed network, which shows an impressive similarity with the Amazon river network (See SI for further comparisons). This similarity allows us to obtain the length of the longest river in a basin by approximating it by the length of the longest path from the point of minimal height (the mouth of the river) of the largest tree on the anti-watershed network. We defined the height of each point of the anti-watershed lines as the mean of the heights of its neighbouring sites. Therefore, we are ignoring any issues related to the initiation point of river channels^57^. As a perspective for future work, quantitative analyses can be performed comparing the anti-watershed and river networks, *e.g*. see the proposed method by Grieve *et al*.^58^.

**Figure 6:**
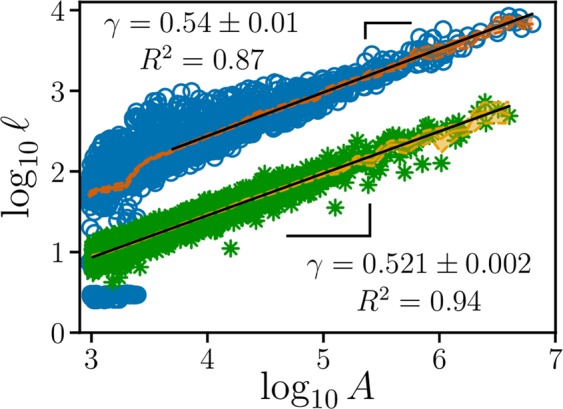
Hack’s law for Earth and for fBm landscapes with *H* = 0.7. Scaling of the longest path $\ell$ from the point of minimal height (mouth) of the largest tree on the anti-watershed network versus the area *A* of its basin (blue circles for Earth and gree asteriks for fBm landscapes), in square kilometres (*km*^2^) for Earth. The solid orange and yellow lines are the Nadaraya-Watson estimator^65,66^, the orange and yellow shaded regions are bounded by the lower and upper confidence intervals, and the solid black line is the linear regression calculated via Ordinary Least Square (OLS)^54^. For Earth, the OLS was applied only to basins greater than 5,000 *km*^2^.

We also performed the verification of Hack’s law^59^ for the entire planet. This scaling law establishes the relation between the areas (*A*) of the basins and the maximum lengths ($\ell$) of their rivers, *i.e*.

1 $$\ell \propto {A}^{\gamma },$$

where *γ* is known as the Hack exponent. In Fig. 6, we show Hack’s law for the original orientation of the terrestrial landscape considering only the basins with area greater than A^*^ = 100 *km*^2^. We chose an area threshold A^*^ to ensure that the analyzed basins were not too much affected by the resolution of the dataset. We found the Hack exponent *γ* = 0.54 ± 0.01 with coefficient of determination *R*^2^ = 0.87, which is very close to our theoretical value of *γ* ≈ 0.55 for Earth.

### Artificial landscapes

We applied the extension of the IPBA to artificial landscapes. In Fig. 3E–H, we show all basins defined by our model for the same samples presented in Fig. 3A–D. As shown in Fig. 4C,D, the perimeter and area distributions obtained from these landscapes are systematically affected by the presence of spatial correlations, quantified here in terms of the parameter *H*. Moreover, except for the case of *H* = −1, all distributions generated from artificial landscapes can be approximately described by power laws, *P*(*s*) ∝ *s*^−*α*^ for perimeters, and *P*(*A*) ∝ *A*^−*β*^ for areas. For each value of *H*, we averaged both distributions for all samples. The insets of the Fig. 4C,D show that *α* and *β* decrease with the Hurst exponent *H*. The exponents *α* and *β* range between 3.36 and 1.93 and between 2.39 and 1.59, respectively. In the uncorrelated case (*H* = −1), however, we obtained less than one order of magnitude for $\ell$ and *A* precluding the same kind of analysis.

In Fig. 6, we show Hack’s law obtained for *H* = 0.7 (a close value of *H* is usually obtained for real landscapes^24^), considering only the basins with area above A^*^ = 1024. Such result led us to the following conjecture: Let the basin area be *A*, the longest anti-watershed line be $\ell$, and assuming that the anti-watershed lines are indeed watershed lines of the upside-down landscapes, it is known that $\ell \propto {L}^{D}$, where *L* is the linear length of the system and *D* is the fractal dimension of the watershed lines^22^. Since *A* ∝ *L*^2^, we have:

2 $${L}^{D}\propto \ell \propto {A}^{\gamma }\propto {L}^{2\gamma },$$

which gives *γ* = *D*/2. In other words, the Hack exponent depends on the fractal dimension of the anti-watershed lines. Fehr *et al*.^24^ showed that the fractal dimension of the watersheds decreases with the Hurst exponent *H*, similarly to the Optimal Path Cracks^31^, and the coastlines on correlated landscapes^60^. The fractal dimension of the watershed lines ranges between *D* = 1.0 and *D* = 1.22, what gives to the Hack exponent a corresponding range from *γ* = 0.5 to *γ* = 0.61. For real landscapes, the fractal dimensions of the watershed lines are around 1.10 (*D* = 1.10 for the Alps^22^, *D* = 1.11 for the Himalaya^22^, and *D* = 1.12 for the Andes^24^). Therefore, our expected Hack exponent for Earth should be *γ* ≈ 0.55, a value which is in good agreement with the result shown in Fig. 6 (*γ* = 0.521 ± 0.002 with coefficient of determination *R*^2^ = 0.94 for artificial landscapes). This result suggests that Hack’s law, often observed for river networks, is an intrinsic effect of topography, *i.e*. it depends, in essence, on the watershed and anti-watershed lines. In other words, Hack’s law may have a purely geometrical origin and does not depend on physical laws governing the water flow on a surface^61^.

## Discussion

We proposed a general model to fully delineate multiple drainage basins for any given landscape of heights through an extension of the IPBA. The novelty of our approach is to characterise all basins from a single height dataset through the definition of a reference (sea) level. Such fact allows us to claim that our model is free of parameter tuning. In this way, we are able to delineate the basins through the definition of the watershed network (maximal lines of a landscape) as well as the anti-basins through the definition of the anti-watershed network (minimal lines of a landscape). In order to show that our algorithm was robust, we applied it to real and artificial landscapes. In both cases, we found that the perimeter and area distributions are ruled by power laws with exponents *α* and *β*, respectively. It was also shown that the terrestrial and martian results are statistically identical, which suggests that the surfaces of Earth and Moon have undergone similar formation processes and that a hypothetical martian river would present similarity to the terrestrial rivers, since both landscapes share the same statistics for watershed and anti-watershed networks. We also verified that, in the Amazon basin and its associated anti-basins defined by our approach, several rivers (the most deeper ones) rest on anti-watershed lines. Furthermore, we showed that the exponents *α* and *β*, for artificial landscapes, decrease systematically with the Hurst exponent *H* and that they are invariant under the inversion of real landscapes. Finally, we found a theoretical value for the Hack’s exponent based on the fractal dimension of the watershed and anti-watershed lines, *γ* = *D*/2. We measured *γ* = 0.521 ± 0.002 for artificial landscapes with *H* = 0.7 and *γ* = 0.54 ± 0.01 for Earth, which agree within error bars with our estimation of *γ* ≈ 0.55 for real cases.

## Methods

### Real landscapes

We use three different DEM datasets throughout this study. The first dataset is the General Bathymetric Chart of the Oceans (GEBCO)^50^ consisting of altimetric and baltimetric heights, *i.e*. the heights above and below the sea level, around the Earth globe. The resolution of this dataset is 30 arc−seconds (30/3600 decimaldegrees) in both coordinates, equivalent to a square lattice with edge length of 0.926 kilometers (*km*) at the Equator line. The other two are the Lunar Orbiter Laser Altimeter (LOLA)^51^ and Mars Orbiter Laser Altimeter (MOLA)^52^ consisting of the map of heights for the Moon and Mars, respectively. The LOLA resolution is about 0.118 *km*, while MOLA resolution is 0.463 *km*, both in relation to their corresponding “Equator”. We show the three datasets in Fig. 2A–C. In addition, we point out that all datasets are freely available and are friendly ready-to-use, *i.e*. all technical preprocessing steps were already performed by the GEBCO, LOLA, and MOLA research teams. We do not perform any additional preprocessing (such as any hydrologic correction or void filling), except the addition of a tiny noise in the heights (<0.001 *m*, much less than the precision of all datasets) in order to ensure that all values in real landscapes are different, defining a unique sequence if the height values are sorted.

We also emphasise that all three datasets are available in the Geographic Coordinate System (GCS), more precisely, in latitude and longitude grids in the image format *TIFF*, *i.e*. they are mapped on spheres, the terrestrial (with radius *R*_*earth*_ = 6378.137 *km*), the lunar (with radius *R*_*moon*_ = 1737.4 *km*), and the martian (with radius *R*_*mars*_ = 3396.19 *km*). For GEBCO, the reference surface (the zero height) is defined by the terrestrial geoid. The geoid is the natural shape that a static fluid would present due to the gravitational potential of its celestial body^62^. On Earth, the oceans could be considered static and, consequently, they are well approximated by such a surface. For LOLA and MOLA, the geoid concept is generalised by the gravitational equipotential surface with the mean lunar and martian radius at the Equator, respectively, defining hypothetical sea levels^51,52^. We adopt the height threshold h^*^ = 0 for all landscapes in order to make a general comparative analysis.

Here, we perform the calculation of the area of each site by the composition of two spherical triangles (the site areas for artificial landscapes have no unit of measure and are all unitary). The area of a spherical triangle with edges *a*, *b* and *c* is given by^63^,

3 $$A=4{R}_{k}^{2}{\tan }^{-1}{[\tan (\frac{s}{2})\tan (\frac{{s}_{a}}{2})\tan (\frac{{s}_{b}}{2})\tan (\frac{{s}_{c}}{2})]}^{1/2},$$

where *s* = (*a*/*R*_*k*_ + *b*/*R*_*k*_ + *c*/*R*_*k*_)/2, *s*_*a*_ = *s* − *a*/*R*_*k*_, *s*_*b*_ = *s* − *b*/*R*_*k*_, and *s*_*c*_ = *s* − *c*/*R*_*k*_. In this formalism, *R*_*k*_ is the sphere radius, where, in our case, *k* = {*earth*, *moon*, *mars*}, and the edge lengths are calculated by the great circle (geodesic) distance between two points *i* and *j* on the sphere surface given by the *Haversine formula*^64^:

4 $${d}_{ij}=2{R}_{k}{\sin }^{-1}[\sqrt{{\sin }^{2}(\frac{{\rm{\Delta }}\varphi }{2})+\,\cos \,{\varphi }_{i}\,\cos \,{\varphi }_{j}{\sin }^{2}(\frac{{\rm{\Delta }}\lambda }{2})}],$$

where Δ*ϕ* = *ϕ*_*j*_ − *ϕ*_*i*_ and Δ*λ* = *λ*_*j*_ − *λ*_*i*_. The values of *λ*_*i*_ (*λ*_*j*_) and *ϕ*_*i*_ (*ϕ*_*j*_), measured in radians, are the longitude and latitude, respectively, of the point *i* (*j*). Therefore, we are able to define the site areas, and, consequently, obtain the total area of a given basin, since each basin is composed by a set of sites.

### Artificial landscapes

We obtained artificial landscapes through the *fractional Brownian motion* (fBm)^53^ in order to study the watershed and anti-watershed networks. One of the most established method to generate a fBm is the so-called *Fourier filtering method* (Ffm)^53^. The basic idea of the Ffm is to define random Fourier coefficients in the reciprocal space, distributed according to the following power-law spectral density:

5 $$S({f}_{1},{f}_{2},\ldots ,{f}_{d})=\sqrt{{(\sum _{i=1}^{d}{f}_{i}^{2})}^{-w}},$$

where *f*_*i*_ is the frequency of the dimension *i*, *d* is the topological dimension, and *w* is the spectral exponent. Subsequently, the inverse Fourier transform is applied to generate a correlated distribution in the real space. In our case *d* = 2, the correlated distribution is a landscape. Each landscape is characterised by an exponent *H*, called *Hurst exponent*, related to the spectral exponent by *w* = 2*H* + *d* = 2*H* + 2. Four cases can be distinguished: (*i*) For *H* = −1, the uncorrelated landscape (see Fig. 3A). (*ii*) For 0 < *H* < 1/2, the landscape has a negative correlation, *i.e*. the increments are anticorrelated (see Fig. 3B). (*iii*) For *H* = 1/2, the landscape is correlated, but the increments are uncorrelated (see Fig. 3C), which is the case of the classical *Brownian motion*^53^. (*iv*) Finally, for 1/2 < *H* < 1, the landscape has a positive correlation, *i.e*. the increments are correlated (see Fig. 3D).

## Supplementary information

Supplementary Information: A universal approach for drainage basins

## Data Availability

All data used in this manuscript are free available. Please, check the references related to GEBCO, LOLA, and MOLA datasets.
