# Supplementary material for: A universal approach for drainage basins
Source: Sci Rep. 2019 Jul 8;9:9845. doi: 10.1038/s41598-019-46165-0 (PMC6614398; doi:10.1038/s41598-019-46165-0)
Supplement: Supplementary file 1 — Supplementary Information: A universal approach for drainage basins [file 41598_2019_46165_MOESM1_ESM.pdf]

Supplementary Information:

A universal approach for drainage basins

Erneson A. Oliveira[1,2,3a], Rilder S. Pires[3], Rubens S. Oliveira[3],
Vasco Furtado[1], Hans J. Herrmann[3,4,5], José S. Andrade Jr.[3]

[1] *Programa de Pós Graduação em Informática Aplicada, Universidade de Fortaleza, 60811-905 Fortaleza, Ceará, Brasil.*

[2] *Mestrado Profissional em Ciências da Cidade, Universidade de Fortaleza, 60811-905 Fortaleza, Ceará, Brasil.*

[3] *Departamento de Física, Universidade Federal do Ceará, Campus do Pici, 60451-970 Fortaleza, Ceará, Brasil.*

[4] *PMMH, ESPCI, 7 quai St Bernard, 75005 Paris, France.*

[5] *ETH Zürich, Computational Physics for Engineering Materials, Institute for Building Materials, Wolfgang-Pauli-Strasse 27, HIT, CH-8093 Zürich, Switzerland*

(Dated: June 26, 2019)

[a] Correspondence to: erneson@eaoliveira.com

I. MAXIMUM LIKELIHOOD ESTIMATOR

Along with the Ordinary Least Square (OLS) [1], we calculated the power law exponents for all distribution present in the main text using the Maximum Likelihood Estimator [2]. Figures 1 and 2 show the Cumulative Distribution Functions (CDFs) for Earth, Moon, and Mars. The Table I summarizes all exponents for real landscapes. Finally, Figure 3 shows the CDF for all values of the Hurst exponent H. Such results lead us to conclude that the MLE leads to similar results of the OLS.

II. THE DEPENDENCY OF THE RESOLUTION FACTOR

To show the possible effects of the resolution factor r in our results, we apply our model to the South America continent (taken from the GEBCO dataset) for the resolutions: $r = 1$ ($\sim 1\ km$), $r = 2$ ($\sim 2\ km$), $r = 4$ ($\sim 4\ km$), and $r = 8$ ($\sim 8\ km$), as ilustrated in Figures 4 and 5. Therefore, we show that the obtained exponents are approximately the same, *i.e.* they are independent of the resolution factor r in the limit of small r. This fact is based on Statistical Physics, where it is known that the effects of finite size are attenuated as the system size increases.

III. ANTI-WATERSHEDS AND HYDROSHEDS DATASET COMPARISON

The anti-watershed and river networks are indeed different objects. An anti-watershed is a watershed on the inverted landscape. In this sense, the anti-watersheds are the minimum lines of that landscape. Therefore, when we join all the anti-watershed lines, we see a network that resembles a river network for the obvious reason that the rivers usually pass through the global minima lines. We show a map with the HydroSHEDS dataset [3] together with the anti-watersheds for the Amazon basin in Figure 6.

[1] Montgomery, D. C., Peck, E. A. & Vining, G. G. *Introduction to linear regression analysis* (John Wiley & Sons, New Jersey, 2012).

[2] Clauset, A., Shalizi, R. C. & Newman, M. E. J. Power-Law Distributions in Empirical Data. *SIAM Review* **51**, 661-703, https://dx.doi.org/10.1137/070710111 (2009).

[3] World Wildlife Fund (WWF) (2006). Hydrological data and maps based on SHuttle Elevation Derivatives at multiple Scales (HydroSHEDS). Available at https://hydrosheds.org/ Accessed April 16, 2019.

Cases	Abbreviation	s_{min}	α	A_{min}	β
Terrestrial Basins	TB	90.43	2.22 ± 0.01	295.32	1.70 ± 0.01
Terrestrial Anti-basins	TA	267.16	2.34 ± 0.02	550.86	1.68 ± 0.01
Lunar Basins	LB	90.56	2.60 ± 0.02	91.43	1.89 ± 0.01
Lunar Anti-basins	LA	115.96	2.76 ± 0.02	415.99	1.97 ± 0.01
Martian Basins	MB	148.19	2.15 ± 0.02	315.84	1.66 ± 0.01
Martian Anti-basins	MA	229.66	2.23 ± 0.03	671.53	1.70 ± 0.01

TABLE I. **Exponents α and β for real landscapes using the Maximum Likelihood Estimator (MLE).** The exponents for area and perimeter distributions of Earth, Moon and Mars obtained with our extension of the Invasion Percolation-Based Algorithm (IPBA).

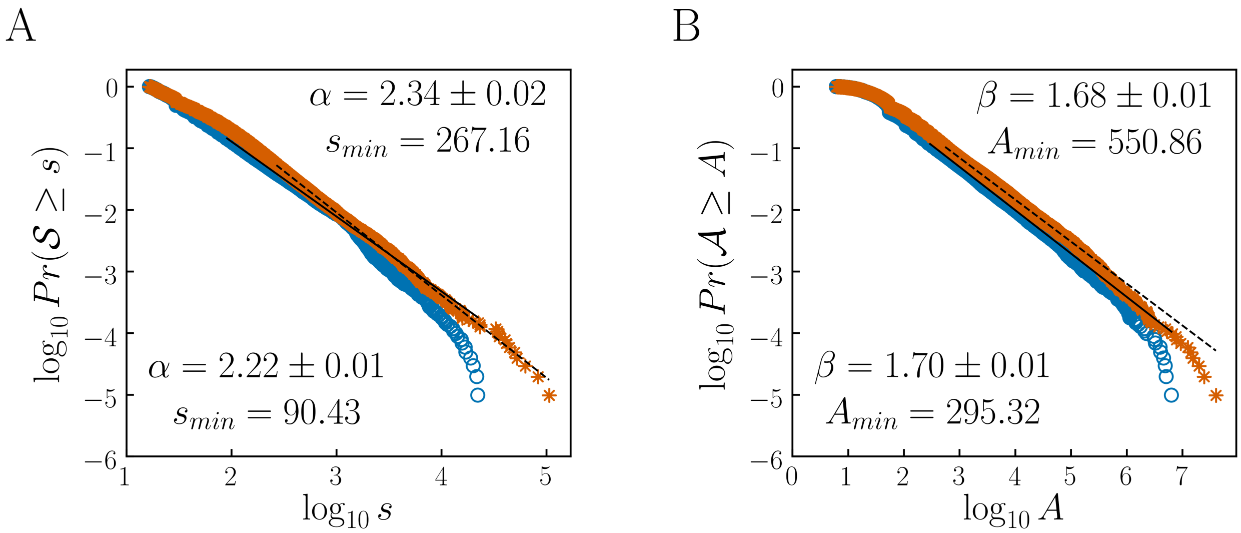

FIG. 1. **(Color online) Cumulative Distribution Functions (CDF), in log–log scale, for basins and anti-basins on Earth.** (a) The perimeter cumulative distribution for the basins (blue circles) and anti-Basins (red asterisks). (b) The area cumulative distribution for the basins (blue circles) and anti-Basins (red asterisks). In both pictures, the solid and dashed lines stand for the power-law fit defined by the Maximum Likelihood Estimator [2] for the basins (lower exponents) and anti-basins (upper exponents). In addition to the MLE exponents (α and β), we also show the values of the lower bounds s_{min} and A_{min}.

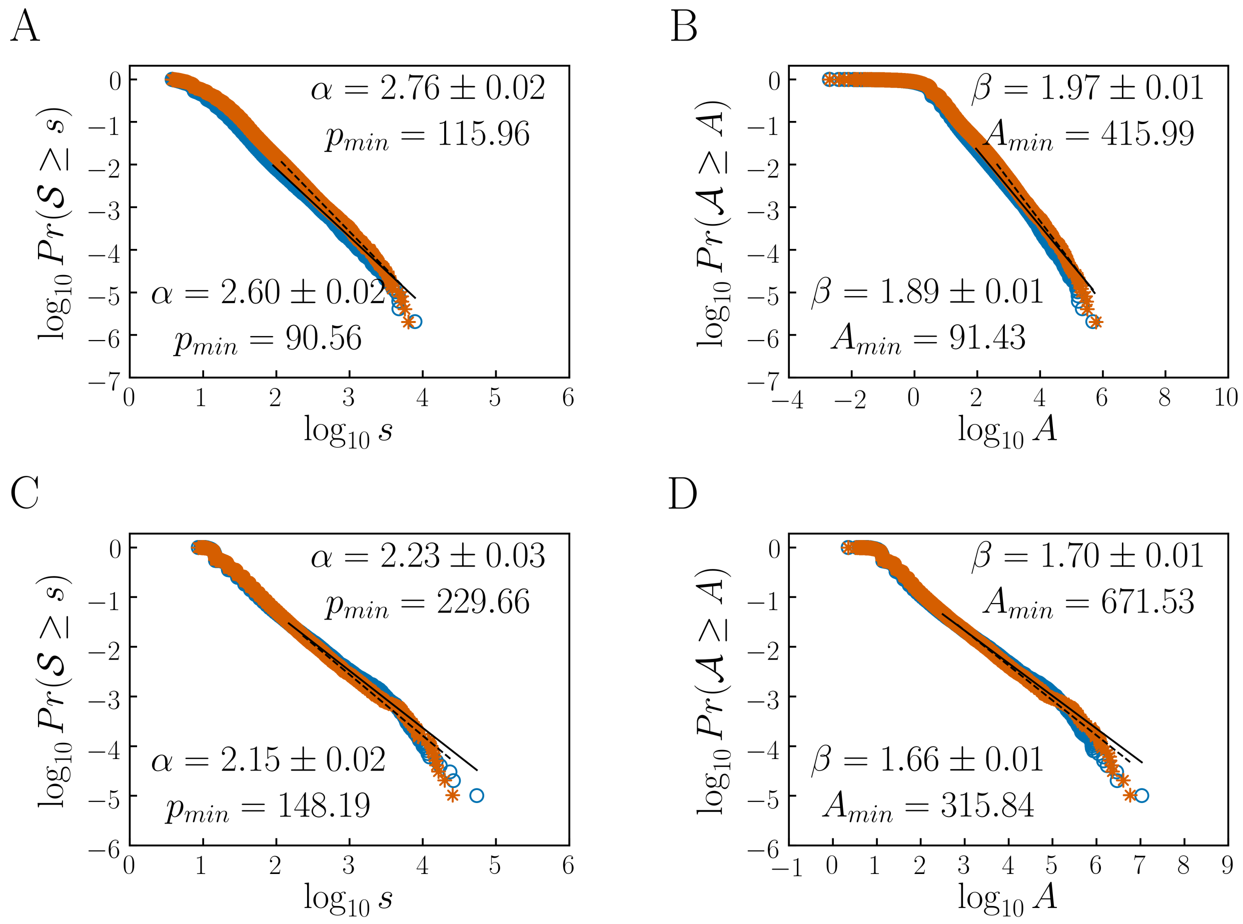

FIG. 2. **(Color online) Cumulative Distribution Functions (CDF), in log–log scale, for basins and anti-basins on Moon and on Mars.** (a) The perimeter cumulative distribution for the basins (blue circles) and anti-Basins (red asterisks). (b) The area cumulative distribution for the basins (blue circles) and anti-Basins (red asterisks). In both pictures, the solid and dashed lines stand for the power-law fit defined by the Maximum Likelihood Estimator [2] for the basins (lower exponents) and anti-basins (upper exponents). In addition to the MLE exponents (α and β), we also show the lower bounds s_{min} and A_{min}.

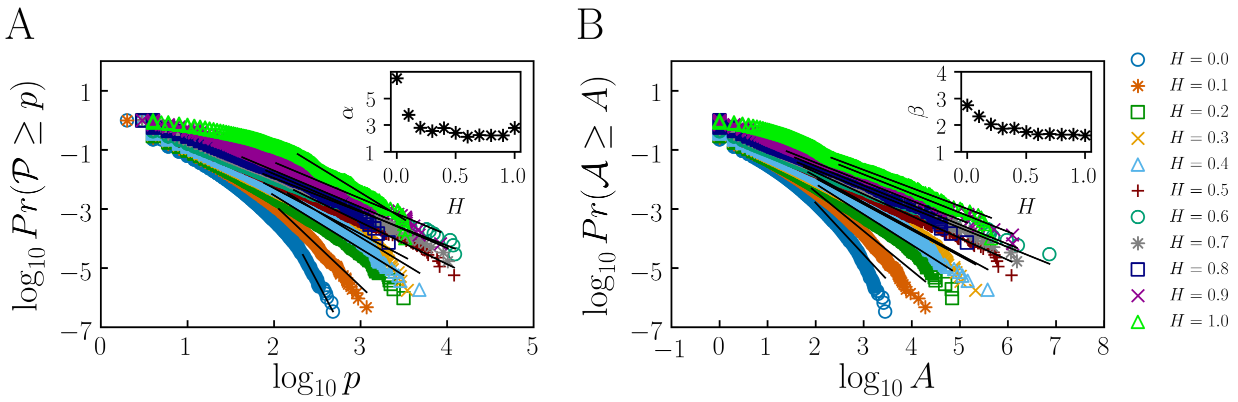

FIG. 3. **(Color online) Cumulative Distribution Functions (CDF), in log–log scale, for basins and anti-basins on fractional Brownian motion (fBm) landscapes.** (a) The perimeter cumulative distribution for several values of the Hurst exponent H. (b) The area cumulative distribution for several values of H. In both plots, the solid lines stand for the power-law fit defined by the Maximum Likelihood Estimator [2]. We also show the behavior of the α and β exponents in the insets.

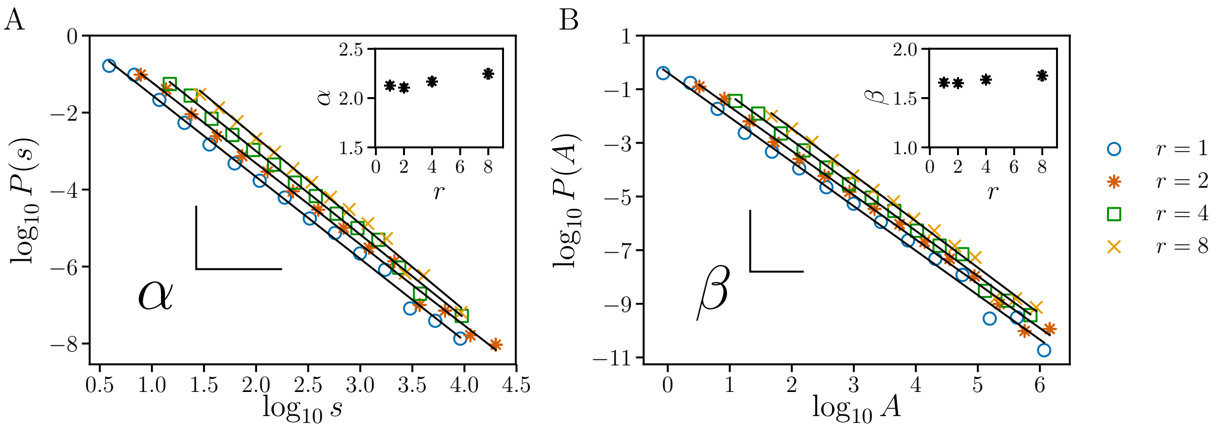

FIG. 4. **(Color online) Log-log plots of the perimeter and area distributions for basins in South America continent.** (A) The perimeter distributions for different values of the resolution factor r. (B) The surface area distributions for different values of resolution factor r. The insets show the behaviour of the exponents α and β as a function of r. All exponents are calculated through the Ordinary Least Square (OLS) fits [1]. We also obtain similar exponents using a Maximum Likelihood Estimator (MLE) [2].

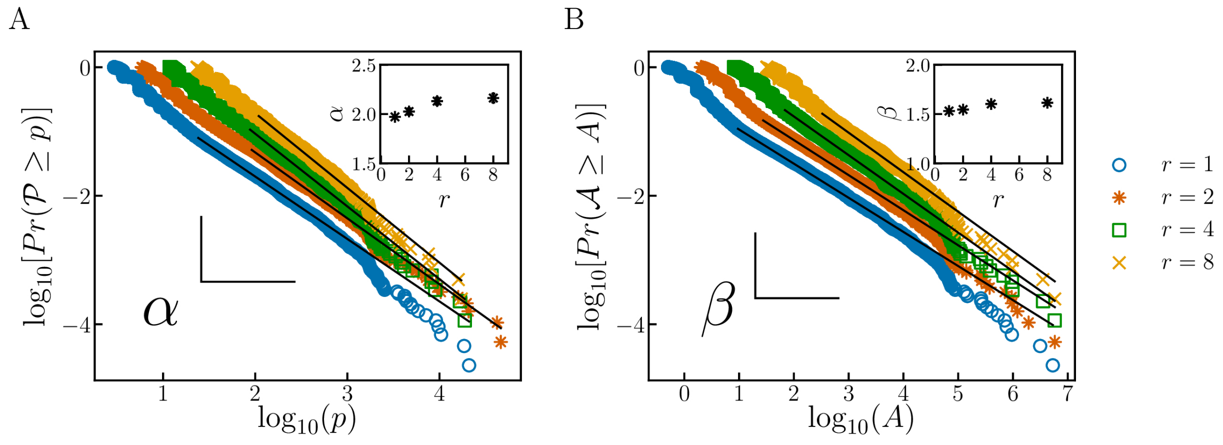

FIG. 5. **(Color online) Cumulative Distribution Functions (CDF), in log–log scale, for basins in South America continent.** (a) The perimeter cumulative distribution for different values of the resolution factor r. (b) The area cumulative distribution for different values of the resolution factor r. In both plots, the solid lines stand for the power-law fit defined by the Maximum Likelihood Estimator [2]. We also show the behavior of the α and β exponents in the insets.

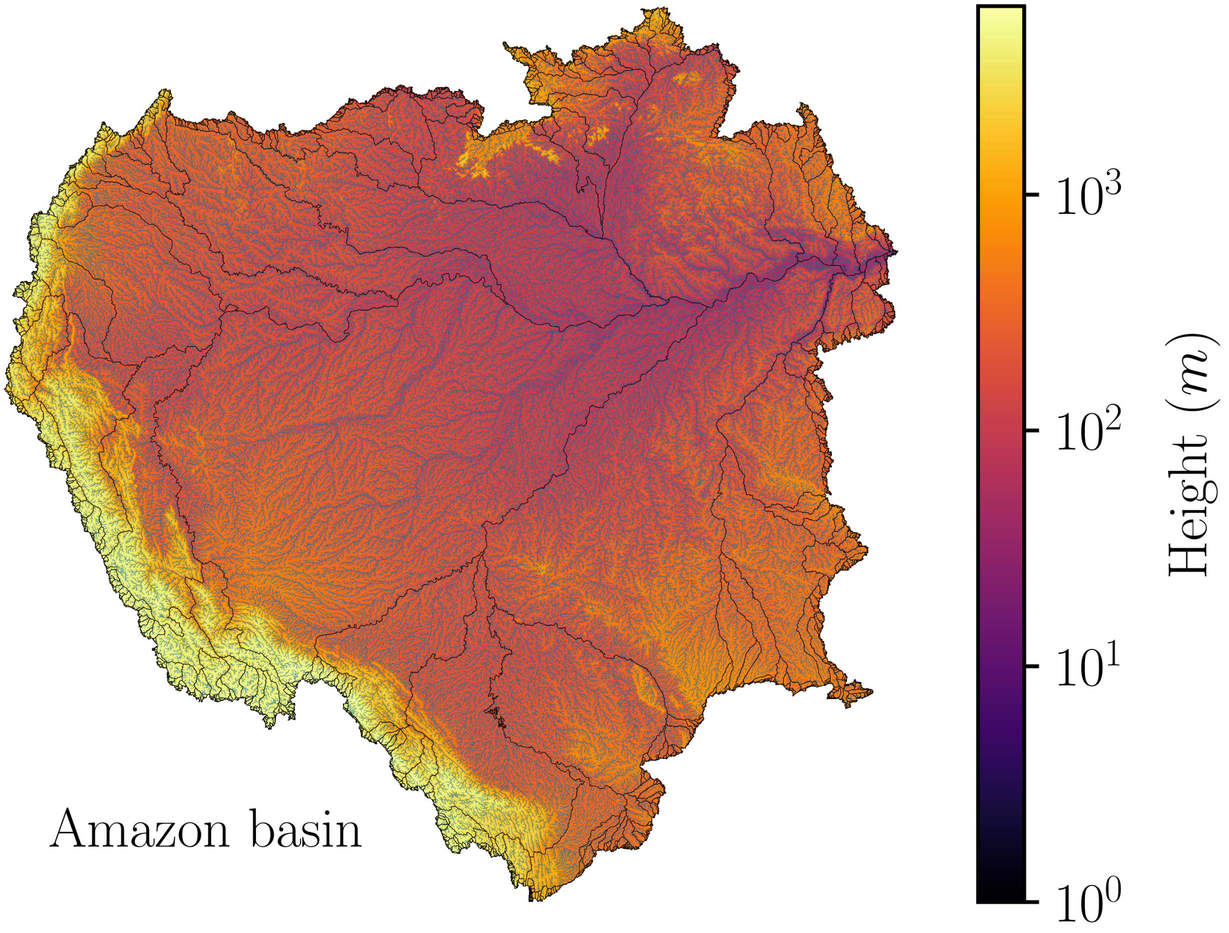

FIG. 6. **The Amazon basin.** The drainage basin of the Amazon region obtained by our algorithm. The heights are represented in metres (m) and in logarithmic scale in order to show the details of the Andes mountain range and the Amazon river. The black lines stand for the anti-watershed network, which shows an impressive similarity with the blue lines that stand for the HydroSHEDs dataset [3]. The resolution of HydroSHEDS is 15 arc-seconds (15/3600 decimal degrees) in both coordinates, equivalent to a square lattice with edge length of 0.463 kilometers (km) at the Equator line.
